# Impact of individual microvascular disease on the risks of macrovascular complications in type 2 diabetes: a nationwide population-based cohort study

**DOI:** 10.1186/s12933-023-01821-8

**Published:** 2023-05-09

**Authors:** Fu-Shun Yen, James Cheng-Chung Wei, Ying-Hsiu Shih, Chih-Cheng Hsu, Chii-Min Hwu

**Affiliations:** 1 Dr Yen’s Clinic, Taoyuan, Taiwan; 2 Department of Allergy, Immunology & Rheumatology, Chung Shan Medical University Hospital, Taichung, Taiwan; 3 Institute of Medicine, Chung Shan Medical University, Taichung, Taiwan; 4 Graduate Institute of Integrated Medicine, China Medical University, Taichung, Taiwan; 5 Management Office for Health Data, China Medical University Hospital, Taichung, Taiwan; 6 Institute of Population Health Sciences, National Health Research Institutes, Miaoli County, Taiwan; 7 Department of Health Services Administration, China Medical University, Taichung, Taiwan; 8 Department of Family Medicine, Min-Sheng General Hospital, Taoyuan, Taiwan; 9 National Center for Geriatrics and Welfare Research, National Health Research Institutes, Yunlin County, Taiwan; 10 Faculty of Medicine, National Yang-Ming Chiao Tung University School of Medicine, Taipei, Taiwan; 11 Section of Endocrinology and Metabolism, Department of Medicine, Taipei Veterans General Hospital, Taipei, Taiwan

**Keywords:** Diabetic kidney disease, Diabetic retinopathy, Diabetic neuropathy, Coronary artery disease, Stroke, Heart failure, Cardiovascular death

## Abstract

**Background:**

This study compared the risks of cardiovascular morbidity and mortality between patients with type 2 diabetes (T2D) with and without microvascular diseases, and between matched patients with microvascular diseases.

**Methods:**

We identified newly diagnosed type 2 diabetes patients from National Health Insurance Research Database in Taiwan from January 1, 2008, to December 31, 2019. Propensity score matching was applied to construct matched pairs of patients with diabetic kidney disease, retinopathy, or neuropathy. Multivariable Cox proportional-hazard models were adopted to compare the risks of cardiovascular morbidity and mortality.

**Results:**

Patients with microvascular disease had a significantly higher risk of cardiovascular morbidities and mortality than those without microvascular disease. Among the matched cohorts, patients with diabetic retinopathy had a significantly higher risk of stroke development than those with diabetic kidney disease (aHR 1.11, 95%CI 1.03–1.2). Diabetic neuropathy showed a significantly higher risk of stroke development than diabetic kidney disease (aHR 1.17, 95%CI 1.1–1.25) and diabetic retinopathy (aHR 1.12, 95%CI 1.03–1.21). Diabetic retinopathy had a significantly higher risk of incident heart failure than diabetic kidney disease (aHR 1.43, 95%CI 1.3–1.57), and diabetic neuropathy had a significantly lower risk of incident heart failure than diabetic retinopathy (aHR 0.79, 95%CI 0.71–0.87).

**Conclusions:**

T2D patients with microvascular disease have a significantly higher risk of cardiovascular morbidities and mortality than those without microvascular disease. In the matched cohorts, diabetic neuropathy was significantly associated with stroke development, and diabetic retinopathy had a significant association with heart failure compared to other microvascular diseases.

**Supplementary Information:**

The online version contains supplementary material available at 10.1186/s12933-023-01821-8.

## Background

According to the International Diabetes Federation (IDF) Diabetes Atlas 2021, there are 537 million adults (20–79 years) with diabetes mellitus (DM) worldwide; this prevalence rate is about 3.56 times that of 2000 (151 million), and the estimated prevalence will jump to a startling 783 million in 2045 [1]. Stroke, myocardial infarction, and heart failure are the main macrovascular complications of diabetes mellitus, which can lead to devastating premature death [2]. Diabetic kidney disease (DKD), retinopathy, and neuropathy are major microvascular complications of diabetes mellitus, which can lead to dialysis, blindness, and amputation [3, 4]. These major microvascular complications can affect the daily life of patients and impose a significant burden on the family and society [3, 4]. In recent years, aggressive glucose control has reduced macrovascular and microvascular diseases in many countries. However, the global burden of macrovascular and microvascular diseases is still alarming owing to improved life expectancy and the increased prevalence of diabetes mellitus [5].

Although there are some cardiovascular prediction models available for patients with type 2 diabetes (T2D), their predictive accuracy is moderate (most < 0.8) [6]. Some patients with diabetes mellitus who do not have a high-risk score still develop macrovascular disease, which suggests that there may be important risk factors not considered in the prediction models [6, 7]. Studies have shown that patients with diabetes mellitus and proteinuria or renal function impairment are prone to cardiovascular morbidity and mortality [3, 8], patients with diabetic retinopathy (DR) are likely to have cardiovascular diseases and heart failure [9], and patients with diabetic neuropathy (DN) are at risk of stroke and limb amputation [8, 10]. A cohort study also showed that patients with type 2 diabetes with more microvascular diseases have a higher associated risk of macrovascular diseases and mortality [7]. Diabetic microvascular diseases may have common pathogenic pathways with the development of macrovascular diseases, but it remains unclear whether the individual microvascular disease has a different association with the development of macrovascular diseases. Therefore, we conducted this nationwide cohort study to compare the risks of macrovascular disease development (1) between patients with type 2 diabetes with and without microvascular disease, (2) between matched patients of diabetic kidney disease versus retinopathy, diabetic kidney disease versus neuropathy, diabetic retinopathy versus neuropathy.

## Methods

### Data source

The Taiwanese government implemented the National Health Insurance (NHI) in 1995 to provide a mandatory comprehensive medical care plan. About 99% of 23 million citizens were enrolled in the NHI program by 2000 [11]. Patient information obtained during medical visits, including age, gender, residential area, diagnosis, procedures, and hospitalizations, is recorded in the National Health Insurance Research Database (NHIRD). Disease diagnosis of the NHIRD is according to the International Classification of Diseases, Ninth/Tenth Revision, Clinical Modification (ICD-9/10-CM). NHIRD has a link with the National Death Registry to substantiate the cause of death. This study identified patients from the NHIRD. All information from patients and care providers was de-identified and encrypted before release to protect individual privacy. This study was approved by the Research Ethics Committee of China Medical University and Hospital (CMUH104-REC2-115-CR4), and the Research Ethics Committee approved a request to waive informed consent.

### Study population and procedures

We identified patients with newly diagnosed type 2 diabetes from January 1, 2009, to December 31, 2018, and followed them up to December 31, 2019. The diagnosis of T2D was based on the ICD codings (Additional file 1: Table S1), with at least 3 outpatient visits within 1 year or one hospitalization [12]. Exclusion criteria were as follows: (1) missing information on age or sex; (2) age below 18 or above 80 years; (3) previous diagnosis of type 2 diabetes before January 1, 2009; (4) diagnosis of type 1 diabetes; (5) diagnosis of chronic kidney disease, dialysis, diabetic retinopathy, or diabetic neuropathy before the diagnosis of T2D; (6) diagnosis of coronary artery disease (CAD), stroke, atrial fibrillation, or heart failure before the index date; (7) death or follow-up for less than 180 days after the index date.

Patients with T2D diagnosis within one year were identified and categorized as (1) without microvascular disease, (2) diabetic kidney disease, including proteinuria, diabetic and hypertensive nephropathy, chronic kidney disease, renal failure, dialysis and renal replacement [13, 14], (3) diabetic retinopathy, including background and proliferative diabetic retinopathy, macular degeneration, retinal edema and detachment, vitreous hemorrhage, and vision loss [15], (4) diabetic neuropathy, including mononeuropathy and polyneuropathy, cranial nerve and peripheral nerve palsy, autonomic neuropathy, neuralgia, and Charcot’s arthropathy [16, 17], (5) diabetic kidney disease and retinopathy, (6) diabetic kidney disease and neuropathy, (7) diabetic retinopathy and neuropathy, (8) diabetic kidney disease, retinopathy, and neuropathy (Additional file 1: Table S2). We defined the 366th day after the diagnosis of type 2 diabetes as the index date of this study and assessed the outcomes during the follow-up period.

### Demographics, comorbidities, and medications

The study included the following variables: age, gender, obesity, smoking, alcohol-related disorders, hypertension, dyslipidemia, peripheral arterial disease (PAD), chronic obstructive pulmonary disease (COPD), liver cirrhosis, connective tissue diseases, cancers, psychosis, depression, dementia, Charlson Comorbidity Index (CCI) score [18], antidiabetic agents, antihypertensive drugs, statin, and aspirin.

### The outcomes of interest

The outcomes of interest were the development of CAD, stroke, heart failure, and cardiovascular death. Stroke, CAD, and heart failure were diagnosed by at least two outpatient visits within 1 year or one hospitalization during the study period [19, 20]. The adjudication of cardiovascular death was via the link with the National Death Registry. Each patient was tracked to the occurrence of outcomes, discontinuation of the NHI program, or the study observation end date of December 31, 2019, depending on which occurred first.

### Statistical analyses

Multivariable-adjusted Cox proportional-hazard models were used to measure the risk of outcomes between patients with and without microvascular disease. Propensity score matching was used to construct matched pairs of patients with diabetic kidney disease and retinopathy, diabetic kidney disease and neuropathy, and diabetic retinopathy and neuropathy. We adopted the non-parsimonious multivariable logistic regression to appraise the propensity score for every patient, and 42 clinically relevant variables (Additional file 1: Tables S4–S6) were determined as independent variables. We adopted the nearest-neighbor algorithm to identify matched pairs and considered a standardized mean difference (SMD) < 0.1 to be a negligible difference between the matched pair of patients. We have performed subgroup analyses to see the influence of one, two or three microvascular diseases on composite major adverse cardiovascular events (CAD, stroke, heart failure and cardiovascular death) in the subgroups of patients with and without PAD, with and without insulin, aspirin and statin use.

The analyzed results were shown as hazard ratios (HRs) and 95% confidence intervals (CIs) for the compared groups. The two-tailed *P* value of less than 0.05 was considered statistically significant. SAS v9.4 (SAS Institute, Inc., Cary, NC, USA) was used for analysis.

## Results

We investigated the occurrence of microvascular disease within one year after the diagnosis of type 2 diabetes. There were 718,059 people without microvascular disease, 45,634 people had diabetic kidney disease, 15,778 had diabetic retinopathy, 51,887 had diabetic neuropathy, 11,763 had diabetic kidney disease and retinopathy, 36,850 had diabetic kidney disease and neuropathy, 10,917 had diabetic retinopathy and neuropathy, 8934 had diabetic kidney disease, retinopathy, and neuropathy (Additional file 1: Table S2). The mean follow-up time was 5.55 years.

**Fig. 1:**
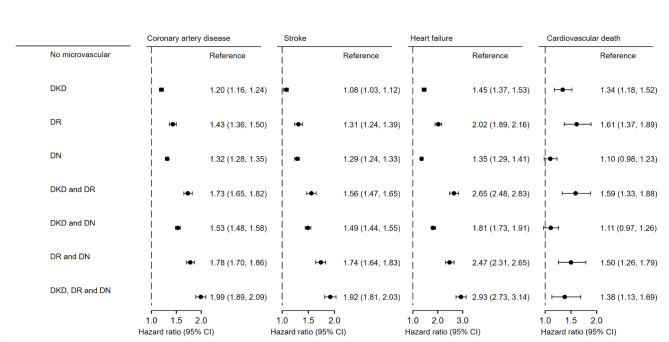
Forest plots of risk for outcomes among patients with and without microvascular diseases. Abbreviations: DKD, diabetes kidney disease; DR, diabetic retinopathy; DN, diabetic neuropathy

Patients with diabetic kidney disease, retinopathy, or neuropathy had a significantly higher risk of coronary artery disease, stroke, heart failure, and cardiovascular death than those without microvascular disease (Fig. 1). Patients with two or three microvascular diseases had a further higher risk of coronary artery disease, stroke, and heart failure than those without microvascular disease (Fig. 1). The subgroup analyses showed that patients with one or two microvascular disease had a significantly higher risk of composite cardiovascular events than those without microvascular disease, which was particularly pronounced in those without PAD compared with those with PAD; people with two or three microvascular diseases had a significantly higher risk of composite cardiovascular events than those with no microvascular disease, which was particularly pronounced in those without aspirin compared with those with aspirin; people with one, two or three microvascular diseases had a significantly higher risk of composite cardiovascular events than those with no microvascular disease, which was further pronounced in those on insulin therapy compared with those not on insulin therapy (P for interaction < 0. 05; Additional file 1: Table S3).

**Fig. 2:**
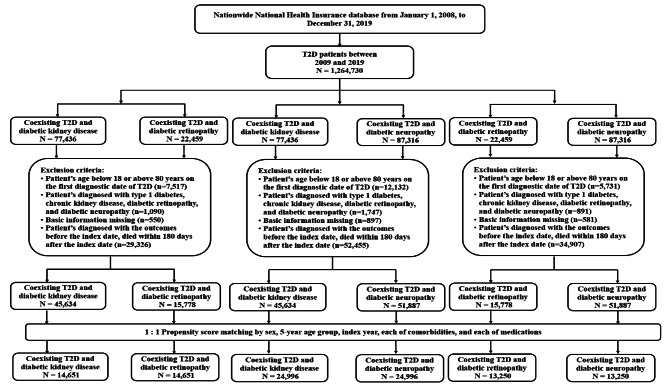
Flowchart for the selection of matched patients

We used propensity-score matching to identify 14,651 pairs of patients with diabetic kidney disease and retinopathy (Fig. 2 and Additional file 1: Table S4), 24,996 pairs of patients with diabetic kidney disease and neuropathy (Additional file 1: Table S5), and 13,250 pairs of patients with diabetic retinopathy and neuropathy (Additional file 1: Table S6). Among the matched cohorts (Table 1), patients with diabetic neuropathy had a significantly higher risk of incident coronary artery disease than those with diabetic kidney disease (aHR 1.07). Patients with diabetic retinopathy had a significantly higher risk of stroke development than those with diabetic kidney disease (aHR 1.11). Diabetic neuropathy showed a significantly higher risk of stroke development than diabetic kidney disease (aHR 1.17). Diabetic neuropathy exhibited a significantly higher risk of stroke development than diabetic retinopathy (aHR 1.12). Diabetic retinopathy had a significantly higher risk of new-onset heart failure than diabetic kidney disease (aHR 1.43). Diabetic neuropathy showed a significantly lower risk of new-onset heart failure than diabetic retinopathy (aHR 0.79).

**Table 1:** Risk for outcomes among matched patients with type 2 diabetes with microvascular diseases

Outcome	Diabetic kidney disease			Diabetic retinopathy								
	n	PY	IR	n	PY	IR	cHR	(95% CI)	p-value	aHR^a^	(95% CI)	p-value
New-onset CAD	1773	75,672	23.43	1869	76,191	24.53	1.05	(0.98, 1.12)	0.1481	1.06	(0.99, 1.13)	0.0777
New-onset stroke	1201	78,169	15.36	1311	78,737	16.65	1.08	(1, 1.17)	0.0416	1.11	(1.03, 1.2)	0.0088
New-onset heart failure	720	80,115	8.99	1012	80,058	12.64	1.41	(1.28, 1.55)	< 0.001	1.43	(1.3, 1.57)	< 0.001
Cardiovascular death	158	82,297	1.92	180	83,122	2.17	1.12	(0.9, 1.39)	0.3033	1.15	(0.93, 1.43)	0.1906
Outcome	Diabetic kidney disease			Diabetic neuropathy								
	n	PY	IR	n	PY	IR	cHR	(95% CI)	p-value	aHR^b^	(95% CI)	p-value
New-onset CAD	2854	122,785	23.24	3062	122,898	24.92	1.07	(1.02, 1.13)	0.0067	1.07	(1.02, 1.13)	0.0064
New-onset stroke	1836	126,841	14.47	2128	126,765	16.79	1.16	(1.09, 1.24)	< 0.001	1.17	(1.1, 1.25)	< 0.001
New-onset heart Failure	1116	129,688	8.61	1110	131,228	8.46	0.98	(0.9, 1.07)	0.6568	1.01	(0.93, 1.1)	0.7501
Cardiovascular death	223	133,095	1.68	195	134,529	1.45	0.85	(0.7, 1.03)	0.0912	0.91	(0.75, 1.1)	0.3205
Outcome	Diabetic retinopathy			Diabetic neuropathy								
	n	PY	IR	n	PY	IR	cHR	(95% CI)	p-value	aHR^c^	(95% CI)	p-value
New-onset CAD	1706	71,331	23.92	1724	70,846	24.33	1.02	(0.95, 1.09)	0.5902	1.02	(0.95, 1.09)	0.5749
New-onset stroke	1173	73,700	15.92	1281	72,661	17.63	1.11	(1.02, 1.2)	0.0114	1.12	(1.03, 1.21)	0.0065
New-onset heart failure	903	74,884	12.06	693	75,471	9.18	0.76	(0.69, 0.84)	< 0.001	0.79	(0.71, 0.87)	< 0.001
Cardiovascular death	152	77,710	1.96	134	77,519	1.73	0.88	(0.7, 1.11)	0.2873	0.91	(0.72, 1.16)	0.4553

Abbreviations: PY, person-years; IR, incidence rate per 1,000 person-years; CAD, coronary artery disease; cHR, crude hazard ratio; aHR, adjusted hazard ratio; adjusted by sex, age, obesity, smoking, alcohol-related disorders, comorbidities, Charlson Comorbidity Index scores, antidiabetic drugs, and cardiovascular-related drugs, as listed in Additional file 1: Tables S4^a^, S5^b^ and S6^c^

**Fig. 3:**
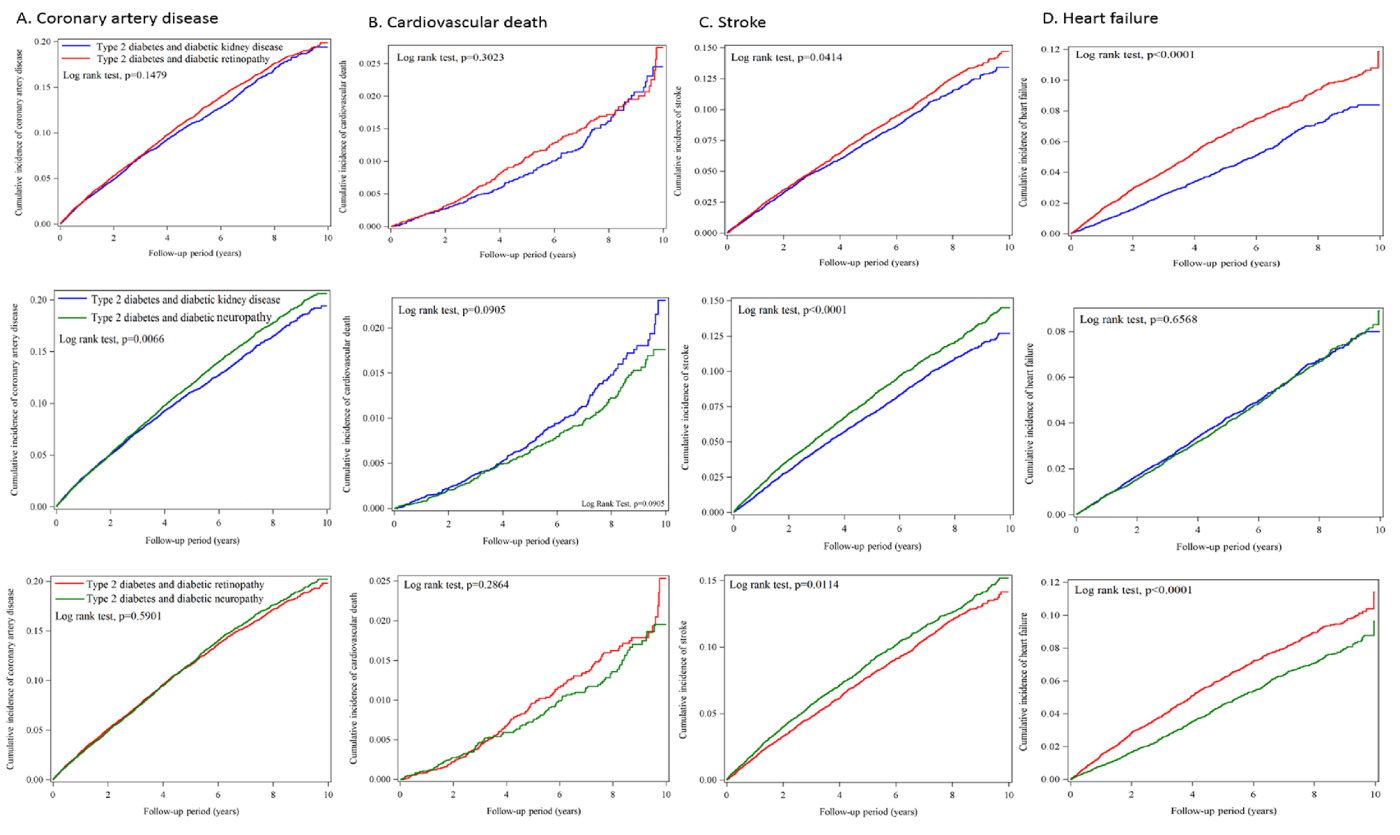
Comparison of the cumulative incidences of macrovascular diseases among the three matched microvascular diseases

In the matched cohorts, diabetic neuropathy had a significantly higher cumulative incidence of incident coronary disease than diabetic kidney disease (Fig. 3). Diabetic neuropathy had a significantly higher cumulative incidence of stroke development than diabetic kidney disease and diabetic retinopathy. Diabetic retinopathy had a significantly higher cumulative incidence of stroke development than diabetic kidney disease. Diabetic retinopathy had a significantly higher cumulative incidence of new-onset heart failure than diabetic kidney disease and diabetic neuropathy.

## Discussion

This nationwide cohort study showed that patients with microvascular disease have a significantly higher risk of cardiovascular disease and mortality than those without microvascular disease, and those with more microvascular diseases have a further higher risk of cardiovascular disease and mortality. In the matched cohorts, the three microvascular diseases had a similar impact on the risk of incident coronary artery disease and cardiovascular mortality; diabetic neuropathy had a higher effect on the risk of stroke than diabetic kidney disease and retinopathy; retinopathy had a higher impact on the development of heart failure than diabetic kidney disease and neuropathy.

Coronary artery disease is the major macrovascular complication in patients with T2D, and about 1–2 in 3 persons with T2D die of cardiovascular disease [2, 21]. Systemic reviews of clinical studies showed that diabetes mellitus with proteinuria increased composite cardiovascular events by about 1.3–4.4 times and cardiovascular death by about 1.2-2.0 times. Renal function impairment increased cardiac events about 2.2 times [8]. Diabetic retinopathy led to a 1.6-4.4-fold increase in the risk of cardiovascular events and a 1.5-5.6-fold increase in the risk of cardiovascular death [8, 9, 22]. Studies on the association between diabetic neuropathy and cardiovascular disease are relatively few. Several studies showed that QT variables were associated with a 0.94-3.3-fold increased risk of cardiovascular death [8]. Brownrigg et al. demonstrated that diabetic nephropathy, retinopathy, and peripheral neuropathy were associated with a consistently higher risk of composite major cardiovascular events and cardiovascular death than no microvascular disease in patients with T2D [7]. Our study also showed that patients with microvascular disease exhibited a significantly higher risk of incident coronary artery disease and cardiovascular death than those without microvascular disease. Patients with more microvascular diseases showed a further higher risk of cardiovascular morbidity and mortality than those with no microvascular disease. The cumulative microvascular diseases may represent a greater generalized vascular abnormality and herald the development of coronary artery disease and cardiovascular death. The impact of the three matched microvascular diseases on coronary artery disease and cardiovascular death seemed similar. Our results are consistent with the results of Brownrigg’s cohort study. Microvascular diseases may provide a more accurate estimation of cardiovascular risk score [7].

In recent years, aggressive glucose control, coronary artery disease, and stroke have gradually decreased, but the incidence of heart failure has increased rather than decreased in the United States [23]. Previous studies revealed that patients with proteinuria exhibited a 1.34-2.45-fold increased risk of heart failure [8]. Patients with diabetic retinopathy showed a 2.71-fold increased risk of heart failure [8, 9]. A large cohort of patients with type 1 diabetes demonstrated that persons with cardiac autonomic neuropathy demonstrated a significantly higher risk of left ventricular dysfunction than those without cardiac autonomic neuropathy [24]. Brownrigg’s cohort study showed that peripheral neuropathy, diabetic nephropathy, and retinopathy were similarly associated with a higher risk of hospitalization for heart failure than those without microvascular disease [7]. The present study showed that patients with the three microvascular diseases exhibited a significantly higher risk of new-onset heart failure than those without microvascular disease, which was consistent with the results of Brownrigg’s study. However, when our three microvascular diseases were matched and compared, we found that diabetic retinopathy had a significantly higher risk of heart failure than diabetic kidney disease and neuropathy, contrary to Brownrigg’s study. The reason could be that our study identified microvascular diseases within one year of T2D diagnosis, but Brownrigg detected microvascular diseases long after T2D diagnosis. The potential explanations for the association of diabetic retinopathy and heart failure are as follows: first, the occurrence of retinopathy may indicate that the dysfunction of coronary microvasculature providing blood circulation to the myocardium causes structural and functional changes in the heart, leading to diabetic cardiomyopathy and heart failure. Studies have demonstrated that a diabetic myocardium is often associated with microaneurysms and perfusion defects [25]. Second, retinopathy may also represent a broad systemic microvascular disease, with increased impedance burden on the heart, compromised cardiac performance, and increased predisposition to heart failure. Patients diagnosed with retinopathy should undergo cardiac function tests to detect heart failure and receive timely preventive treatment.

Patients with T2D have two times higher risk of stroke than those without T2D [26]. Clinical studies showed that patients with diabetes mellitus and nephropathy had a 1.7–2.3 times higher risk of stroke [8]. Patients with retinopathy had a 1.11–7.35 times higher risk of stroke [21]. Patients with cardiac autonomic neuropathy exhibited a 1.7–2.7 folds higher risk of stroke than those without cardiac autonomic neuropathy [8, 10, 27, 28]. Our study showed that patients with microvascular disease demonstrated a significantly higher risk of stroke development than those without microvascular disease. After propensity score matching of the three microvascular diseases, our study indicated that diabetic neuropathy had a higher impact on stroke development than retinopathy and nephropathy. Diabetic neuropathy is a syndrome that includes somatic and autonomic neuropathy of the peripheral nervous system [4, 29]. The plausible mechanisms to explain the intense association of neuropathy and stroke development are as follows: first, diabetic neuropathy may lead to the dysfunction of the parasympathetic and sympathetic innervation of cerebral vasculatures, resulting in the defective autoregulation of cerebral blood flow and render the brain vessels more vulnerable to injury and occlusion [10, 27, 28]. Second, neuropathy may cause unstable baroreflex and heart rhythms and favor atrial fibrillation with subsequent stroke development [28].

This study also has some limitations. First, this dataset lacked information on renal function, proteinuria, blood glucose, biochemical, neurological, fundus, imaging, and pathology findings. Although previous studies validated the algorithm for using ICD codes to define vascular diseases, it may have been unable to detect minor and early vascular lesions, leading to underestimation. Second, this research lacked complete information on dietary habits, exercise, family history, and alcohol consumption, which may have affected the results of this study. However, baseline demographics, obesity, smoking, alcohol-related disorders, comorbidities, antidiabetic drugs, and cardiovascular drugs were well-matched to maximally balance the variables of patients without and with various microvascular diseases and increase comparability. We used comorbidities, CCI scores, insulin, number of oral antidiabetic and antihypertensive drugs as surrogate markers of diabetes severity. Third, a retrospective cohort study cannot exclude unmeasured or unknown residual confounding, and rigorous prospective studies are warranted to justify our results.

## Conclusions

Our study shows that microvascular diseases early in the diagnosis of type 2 diabetes are closely associated with the development of coronary artery disease, stroke, heart failure, and cardiovascular death. Furthermore, people with one, two or three microvascular diseases signify an even higher risk of cardiovascular morbidity and mortality than those without microvascular disease. We also found that these three microvascular diseases may be similarly associated with coronary artery diseases and cardiovascular death; but diabetic retinopathy was significantly associated with new-onset heart failure, and diabetic neuropathy had a significant association with stroke development. Further research may be needed to elucidate whether there are causal relationships and plausible mechanisms between retinopathy and heart failure, neuropathy and stroke.

## Electronic supplementary material

Below is the link to the electronic supplementary material.

Supplementary Material 1

## Data Availability

Data of this study are available from the National Health Insurance Research Database (NHIRD) published by Taiwan National Health Insurance (NHI) Administration. The data utilized in this study cannot be made available in the paper, the supplemental files, or in a public repository due to the ‘‘Personal Information Protection Act’’ executed by Taiwan government starting from 2012. Requests for data can be sent as a formal proposal to the NHIRD Office (https://dep.mohw.gov.tw/DOS/cp-2516-3591-113.html) or by email to stsung@mohw.gov.tw.

## Abbreviations

T2D: type 2 diabetes
DM: diabetes mellitus
DKD: Diabetic kidney disease
DR: diabetic retinopathy
DN: diabetic neuropathy
CAD: coronary artery disease
PAD: peripheral arterial disease
COPD: chronic obstructive pulmonary disease
