# Supplementary material for: Impact of individual microvascular disease on the risks of macrovascular complications in type 2 diabetes: a nationwide population-based cohort study
Source: Cardiovasc Diabetol. 2023 May 9;22:109. doi: 10.1186/s12933-023-01821-8 (PMC10170797; doi:10.1186/s12933-023-01821-8)
Supplement: Supplementary file 1 — Supplementary Material 1 [file 12933_2023_1821_MOESM1_ESM.docx]

**Table S1** Diseases and related ICD-9-CM, ICD-10-CM codes

| **Disease** | **ICD-9-CM codes** | **ICD-10-CM codes** |
| --- | --- | --- |
| Type 2 diabetes | 250.xx, except 250.1x | E11 |
|  |  |  |
| Type 1 diabetes | 250.1x | E10 |
| Coronary artery disease | 398.91, 402, 404, 410-414, ICD-9-CM Procedure code (ICD-9-PCS): 36 | I09.81, I11, I13, I20-I22, I24, I25.1-I25.7, I25.81, I25.89, ICD-10 Procedure code (ICD-10-PCS): 02 |
| Stroke | 430-438 | G45.0, G45.1, G45.2, G45.3, G45.4, G45.8, G45.9, G46, I60, I61, I62, I63, I65, I66, I67.0, I67.1, I67.2, I67.3, I67.4, I67.5, I67.6, I67.7, I67.8, I67.9, I68, I69 |
| Heart failure | 428 | I50 |
| Atrial fibrillation | 427 | I45.0, I45.1, I45.2, I45.3, I45.4, I45.5, I45.6 |
| Cardiovascular death | 390-392、393-398、410-414、420-4299, 430-438 | I01, I02.0, I05-I09, I20-I25, I27, I30-I52, I60-I69 |
| Chronic kidney disease | 250.4, 403-404, 585-586, 581.8, 593.9, 791.0, V42.0, V45.1, V56.0, V56.8 , ICD-9-PCS: 39.27, 39.42-39.43, 39.49-39.50, 39.53, 39.93-39.95 | I12, I13, N08, N18, N19, N29, E10.2, E11.2, E13.2, N02.8, N04.7, N04.8, N18.9, N28.9, R80.8, R80.9, N18.1-N18.3, R80.0-R80.3, Z94.0, Z99.2, Z94.0, E10.65, E11.65, Z49.31, Z49.32 |
| Diabetic retinopathy | 362.01、362.02、362.07, 362.53、362.83、364.42、379.23、369, ICD-9-PCS:14.2-14.5、14.7 | E11.31-E11.34, E11.35, H54, H43.1, H35.35, H35.81, H21.1X, ICD-10-PCS: 085F3ZZ, 08H031Z, 08H0X1Z, 08H131Z, 08H1X1Z |
| Diabetic neuropathy | 250.6, 302.72, 337.0, 337.1, 354-355, 356.9, 357.2, 358.1, 458, 536.3, 564.5, 595.54, 607.84, 713.5, 729.2, 951.0, 951.1, 951.3 | E08-11, E13.49, F52.21, F52.22, F52.8, G56-59, G60.9, G70.8, G70.81, G73.1, G73.3, G90.01, G90.09, G99.0, I95, K59.1, K31.84, M14, M54.10, M54.18, M79.2, N31.0, N31.1, N31.9, N52, S04 |
| Obesity | 278.02, 783.1, V85.2, 278.00, 649.1, V77.8, V85.3, 278.01, 649.2, V45.86, V85.4 | R63.5, E66.09, E66.1, E66.8, E66.9, Z13.89, E66.01, E66.2 |
| Smoking status | 305.1, 649.0, V15.82 | F17.200, F17.201, F17.210, F17.220, F17.221, F17.290, F17.291, Z87.891 |
| Alcohol-related disorders | 291, 303, 305.0, 571.0-571.3, V11.3, V79.1 | F10, K70.40, K70.41, K70.9 |
| Hypertension | 401–405, A26 | I10, I11, I12, I13, I15, N26 |
| Dyslipidemia | 272 | E71.30, E71.31, E71.32, E71.39, E75.21, E75.22, E75.23, E75.24, E75.25, E75.29, E75.3, E75.4, E75.5, E75.6, E77, E78.0, E78.1, E78.2, E78.3, E78.4, E78.5, E78.6, E78.70, E78.71, E78.72, E78.79, E78.8, E78.9 |
| Peripheral arterial disease | 440.0, 440.20, 440.21, 440.22, 440.23, 440.24, 440.3, 440.4, 443.9, 443.81, 443.89 | I70.2, I70.92, I75.0, I73.9 |
| Chronic obstructive pulmonary disease | 491, 492, or 496 | J41, J42, J44, J43, or J44.9 |
|  |  |  |
| Liver cirrhosis | 571.5, 571.2, 571.6 | K70.2, K70.30, K70.31, K74.0, K74.1, K74.2, K74.60, K74.69, K74.3, K74.4, K74.5 |
| Connective tissue diseases | 710.0, 714.0, 725.0 | M32.10, M06.9, M35.3 |
| Cancers | 140-178, 190-199, 209 | C00-C63, C69-C80, C7A-C7B |
| Psychosis | 290–299 | F20-29 |
| Depression | 311 | F32, F33 |
| Dementia | 290,290.4, 291.2, 292.82, 331 | F03.90, F05, F02.80, F02.81, F01.50, F01.51, G30 |

**Table S2** Characteristics of patients with type 2 diabetes with and without microvascular diseases

| **Variables** | **No microvascular complications** | | **Diabetic kidney disease** | | **Diabetic retinopathy** | | **Diabetic neuropathy** | | **DKD+DR** | | **CKD+DN** | | **DR+DN** | | **CKD+DR+DN** | |
| --- | --- | --- | --- | --- | --- | --- | --- | --- | --- | --- | --- | --- | --- | --- | --- | --- |
| Number | (N=718059) | | (N=45634) | | (N=15778) | | (N=51887) | | (N=11763) | | (N=36850) | | (N=10917) | | (N=8934) | |
| Starting year | n | % | n | % | n | % | n | % | n | % | n | % | n | % | n | % |
| 2010 | - | - | 3528 | 7.73 | 2941 | 18.64 | 9163 | 17.66 | 2376 | 20.2 | 5955 | 16.16 | 2785 | 25.51 | 2242 | 25.1 |
| 2011 | - | - | 3255 | 7.13 | 2108 | 13.36 | 6616 | 12.75 | 1693 | 14.39 | 4206 | 11.41 | 1832 | 16.78 | 1471 | 16.47 |
| 2012 | - | - | 4202 | 9.21 | 1896 | 12.02 | 5471 | 10.54 | 1645 | 13.98 | 3847 | 10.44 | 1464 | 13.41 | 1202 | 13.45 |
| 2013 | - | - | 4155 | 9.11 | 1736 | 11 | 4803 | 9.26 | 1375 | 11.69 | 3300 | 8.96 | 1156 | 10.59 | 959 | 10.73 |
| 2014 | - | - | 4321 | 9.47 | 1670 | 10.58 | 4257 | 8.2 | 1233 | 10.48 | 2900 | 7.87 | 1018 | 9.32 | 858 | 9.6 |
| 2015 | - | - | 4083 | 8.95 | 1476 | 9.35 | 3649 | 7.03 | 1034 | 8.79 | 2469 | 6.7 | 842 | 7.71 | 693 | 7.76 |
| 2016 | - | - | 8359 | 18.32 | 1442 | 9.14 | 7171 | 13.82 | 1168 | 9.93 | 5895 | 16 | 887 | 8.12 | 756 | 8.46 |
| 2017 | - | - | 6759 | 14.81 | 1267 | 8.03 | 5450 | 10.5 | 704 | 5.98 | 4237 | 11.5 | 535 | 4.9 | 431 | 4.82 |
| 2018 | - | - | 6972 | 15.28 | 1242 | 7.87 | 5307 | 10.23 | 535 | 4.55 | 4041 | 10.97 | 398 | 3.65 | 322 | 3.6 |
| Sex |  |  |  |  |  |  |  |  |  |  |  |  |  |  |  |  |
| Female | 319443 | 44.49 | 18300 | 40.10 | 7020 | 44.49 | 23491 | 45.27 | 4979 | 42.33 | 15627 | 42.41 | 4888 | 44.77 | 3861 | 43.22 |
| Male | 398616 | 55.51 | 27334 | 59.90 | 8758 | 55.51 | 28396 | 54.73 | 6784 | 57.67 | 21223 | 57.59 | 6029 | 55.23 | 5073 | 56.78 |
| Age |  |  |  |  |  |  |  |  |  |  |  |  |  |  |  |  |
| 18-40 | 101552 | 14.14 | 6563 | 14.38 | 1697 | 10.76 | 5931 | 11.43 | 1328 | 11.29 | 4868 | 13.21 | 1103 | 10.10 | 996 | 11.15 |
| 41-60 | 404213 | 56.29 | 24735 | 54.20 | 9239 | 58.56 | 30051 | 57.92 | 6927 | 58.89 | 20652 | 56.04 | 6574 | 60.22 | 5398 | 60.42 |
| 61-80 | 212294 | 29.57 | 14336 | 31.42 | 4842 | 30.69 | 15905 | 30.65 | 3508 | 29.82 | 11330 | 30.75 | 3240 | 29.68 | 2540 | 28.43 |
| Mean, (SD)^a^ | 53.84 | 12.00 | 54.06 | 12.23 | 54.62 | 10.96 | 54.58 | 11.42 | 54.39 | 11.04 | 54.2 | 11.89 | 54.64 | 10.70 | 54.22 | 10.86 |
| Obesity | 20215 | 2.82 | 1621 | 3.55 | 292 | 1.85 | 1413 | 2.72 | 223 | 1.90 | 1192 | 3.23 | 178 | 1.63 | 151 | 1.69 |
| Smoking | 14818 | 2.06 | 1463 | 3.21 | 307 | 1.95 | 1529 | 2.95 | 210 | 1.79 | 1149 | 3.12 | 197 | 1.80 | 158 | 1.77 |
| Comorbidities |  |  |  |  |  |  |  |  |  |  |  |  |  |  |  |  |
| Hypertension | 357916 | 49.84 | 26838 | 58.81 | 8758 | 55.51 | 26361 | 50.80 | 6839 | 58.14 | 19757 | 53.61 | 5703 | 52.24 | 4797 | 53.69 |
| Dyslipidemia | 432012 | 60.16 | 32369 | 70.93 | 9831 | 62.31 | 32521 | 62.68 | 7452 | 63.35 | 23991 | 65.10 | 6536 | 59.87 | 5391 | 60.34 |
| Peripheral arterial disease | 9732 | 1.36 | 883 | 1.94 | 367 | 2.33 | 1606 | 3.10 | 294 | 2.50 | 1016 | 2.76 | 356 | 3.26 | 282 | 3.16 |
| COPD | 109797 | 15.29 | 7329 | 16.06 | 1808 | 11.46 | 9209 | 17.75 | 1344 | 11.43 | 6242 | 16.94 | 1311 | 12.01 | 1063 | 11.90 |
| Alcohol-related disorders | 17500 | 2.44 | 1324 | 2.90 | 275 | 1.74 | 1669 | 3.22 | 210 | 1.79 | 1221 | 3.31 | 236 | 2.16 | 186 | 2.08 |
| Liver cirrhosis | 11826 | 1.65 | 959 | 2.10 | 219 | 1.39 | 1012 | 1.95 | 157 | 1.33 | 735 | 1.99 | 148 | 1.36 | 117 | 1.31 |
| Connective tissue diseases | 12861 | 1.79 | 1024 | 2.24 | 198 | 1.25 | 1416 | 2.73 | 140 | 1.19 | 851 | 2.31 | 161 | 1.47 | 124 | 1.39 |
| Cancers | 31930 | 4.45 | 2523 | 5.53 | 546 | 3.46 | 2577 | 4.97 | 403 | 3.43 | 1806 | 4.90 | 365 | 3.34 | 296 | 3.31 |
| Psychosis | 33871 | 4.72 | 2276 | 4.99 | 531 | 3.37 | 3087 | 5.95 | 373 | 3.17 | 2114 | 5.74 | 390 | 3.57 | 307 | 3.44 |
| Depression | 9783 | 1.36 | 649 | 1.42 | 156 | 0.99 | 1025 | 1.98 | 104 | 0.88 | 631 | 1.71 | 122 | 1.12 | 93 | 1.04 |
| Dementia | 3670 | 0.51 | 279 | 0.61 | 50 | 0.32 | 364 | 0.70 | 36 | 0.31 | 248 | 0.67 | 37 | 0.34 | 32 | 0.36 |
| CCI |  |  |  |  |  |  |  |  |  |  |  |  |  |  |  |  |
| 1 | 657913 | 91.62 | 38403 | 84.15 | 13309 | 84.35 | 44469 | 85.70 | 9805 | 83.35 | 30801 | 83.58 | 9165 | 83.95 | 7464 | 83.55 |
| 2-3 | 53119 | 7.40 | 5811 | 12.73 | 2074 | 13.14 | 6125 | 11.80 | 1670 | 14.20 | 5032 | 13.66 | 1493 | 13.68 | 1273 | 14.25 |
| >3 | 7027 | 0.98 | 1420 | 3.11 | 395 | 2.50 | 1293 | 2.49 | 288 | 2.45 | 1017 | 2.76 | 259 | 2.37 | 197 | 2.21 |
| Medications |  |  |  |  |  |  |  |  |  |  |  |  |  |  |  |  |
| Antidiabetic drugs |  |  |  |  |  |  |  |  |  |  |  |  |  |  |  |  |
| Metformin | 259742 | 36.17 | 29267 | 64.13 | 11750 | 74.47 | 26625 | 51.31 | 8671 | 73.71 | 22199 | 60.24 | 7570 | 69.34 | 6275 | 70.24 |
| Sulfonylurea | 152123 | 21.19 | 16496 | 36.15 | 8433 | 53.45 | 16802 | 32.38 | 6701 | 56.97 | 14117 | 38.31 | 5925 | 54.27 | 5002 | 55.99 |
| Meglitinides | 9214 | 1.28 | 1393 | 3.05 | 695 | 4.40 | 1266 | 2.44 | 561 | 4.77 | 1118 | 3.03 | 466 | 4.27 | 383 | 4.29 |
| Thiazolidinedione | 12449 | 1.73 | 1567 | 3.43 | 946 | 6.00 | 1599 | 3.08 | 775 | 6.59 | 1341 | 3.64 | 671 | 6.15 | 566 | 6.34 |
| DPP-4 inhibitors | 32820 | 4.57 | 6631 | 14.53 | 2453 | 15.55 | 5109 | 9.85 | 1947 | 16.55 | 4737 | 12.85 | 1564 | 14.33 | 1330 | 14.89 |
| AGI | 15595 | 2.17 | 1958 | 4.29 | 1025 | 6.50 | 1820 | 3.51 | 805 | 6.84 | 1583 | 4.30 | 677 | 6.20 | 570 | 6.38 |
| SGLT2 inhibitors | 1305 | 0.18 | 406 | 0.89 | 79 | 0.50 | 291 | 0.56 | 50 | 0.43 | 249 | 0.68 | 38 | 0.35 | 34 | 0.38 |
| Number of oral antidiabetic drugs | | |  |  |  |  |  |  |  |  |  |  |  |  |  |  |
| 1 | 566336 | 78.87 | 26409 | 57.87 | 6572 | 41.65 | 33749 | 65.04 | 4539 | 38.59 | 21301 | 57.80 | 4647 | 42.57 | 3651 | 40.87 |
| 2-3 | 149204 | 20.78 | 18719 | 41.02 | 8861 | 56.16 | 17658 | 34.03 | 6952 | 59.10 | 15111 | 41.01 | 6036 | 55.29 | 5083 | 56.90 |
| >3 | 2519 | 0.35 | 506 | 1.11 | 345 | 2.19 | 480 | 0.93 | 272 | 2.31 | 438 | 1.19 | 234 | 2.14 | 200 | 2.24 |
| GLP-1 RA | 226 | 0.03 | 96 | 0.21 | 29 | 0.18 | 62 | 0.12 | 14 | 0.12 | 62 | 0.17 | 12 | 0.11 | 10 | 0.11 |
| Insulins | 12111 | 1.69 | 3119 | 6.83 | 1481 | 9.39 | 2700 | 5.20 | 1279 | 10.87 | 2668 | 7.24 | 1109 | 10.16 | 994 | 11.13 |
| Antihypertensive drugs | |  |  |  |  |  |  |  |  |  |  |  |  |  |  |  |
| ACEI/ARB | 148290 | 20.65 | 15067 | 33.02 | 5075 | 32.17 | 11348 | 21.87 | 4133 | 35.14 | 9472 | 25.70 | 2963 | 27.14 | 2577 | 28.84 |
| β-blockers | 14025 | 1.95 | 799 | 1.75 | 239 | 1.51 | 1151 | 2.22 | 157 | 1.33 | 747 | 2.03 | 166 | 1.52 | 117 | 1.31 |
| Calcium-channel blockers | 119010 | 16.57 | 8028 | 17.59 | 2914 | 18.47 | 8117 | 15.64 | 2196 | 18.67 | 6003 | 16.29 | 1808 | 16.56 | 1473 | 16.49 |
| Diuretics | 22921 | 3.19 | 2143 | 4.70 | 975 | 6.18 | 1749 | 3.37 | 762 | 6.48 | 1441 | 3.91 | 559 | 5.12 | 467 | 5.23 |
| Number of antihypertensive drugs | | |  |  |  |  |  |  |  |  |  |  |  |  |  |  |
| 1 | 648679 | 90.34 | 39882 | 87.40 | 13534 | 85.78 | 47042 | 90.66 | 9965 | 84.71 | 32940 | 89.39 | 9595 | 87.89 | 7814 | 87.46 |
| 2-3 | 69281 | 9.65 | 5742 | 12.58 | 2236 | 14.17 | 4837 | 9.32 | 1794 | 15.25 | 3906 | 10.60 | 1318 | 12.07 | 1120 | 12.54 |
| >3 | 99 | 0.01 | 10 | 0.02 | 8 | 0.05 | 8 | 0.02 | 4 | 0.03 | 4 | 0.01 | 4 | 0.04 |  |  |
| Statin | 122104 | 17.00 | 13173 | 28.87 | 4653 | 29.49 | 10480 | 20.20 | 3464 | 29.45 | 8586 | 23.30 | 2676 | 24.51 | 2193 | 24.55 |
| Aspirin | 27088 | 3.77 | 2225 | 4.88 | 896 | 5.68 | 2269 | 4.37 | 693 | 5.89 | 1710 | 4.64 | 581 | 5.32 | 490 | 5.48 |

Data are shown as n (%) or mean ± SD

Abbreviations: DKD, diabetes kidney disease; DR, diabetic retinopathy; DN, diabetic neuropathy; COPD, chronic obstructive pulmonary disease; CCI, Charlson Comorbidity Index; DPP-4, Dipeptidyl peptidase-4; AGI, Alpha-glucosidase inhibitors; SGLT2, sodium-glucose cotransporter 2; OAD, oral antidiabetic drug; GLP-1RA, glucagon-like peptide-1 receptor agonist; ACEI, angiotensin converting enzyme inhibitors; ARB, angiotensin receptor blockers

^a^: Student’s t-test

| **Table S3** Incidences and hazard ratios of MACE for patients with and without microvascular complications stratified by variables. | | | | | | | | | | | |
| --- | --- | --- | --- | --- | --- | --- | --- | --- | --- | --- | --- |
|  | **Without microvascular disease** | | | **With one microvascular disease** | | |  |  |  |  | **P for interaction** |
|  | **Event** | **PY** | **IR** | **Event** | **PY** | **IR** | **cHR** | **(95% CI)** | **aHR^#^** | **(95% CI)** |  |
| Overall | 119816 | 3829810 | 31.29 | 18675 | 450770 | 41.43 | 1.32 | (1.30, 1.34)*** | 1.25 | (1.23, 1.28)*** |  |
| Peripheral arterial disease |  |  |  |  |  |  |  |  |  |  | 0.0441 |
| No | 117507 | 3782964 | 31.06 | 18017 | 439467 | 41.00 | 1.32 | (1.29, 1.34)*** | 1.26 | (1.24, 1.28)*** |  |
| Yes | 2309 | 46846 | 49.29 | 658 | 11304 | 58.21 | 1.18 | (1.08, 1.29)*** | 1.18 | (1.07, 1.30)*** |  |
| Insulin use |  |  |  |  |  |  |  |  |  |  | <.0001 |
| No | 118389 | 3768756 | 31.41 | 17647 | 428582 | 41.18 | 1.31 | (1.29, 1.33)*** | 1.25 | (1.22, 1.27)*** |  |
| Yes | 1427 | 61054 | 23.37 | 1028 | 22189 | 46.33 | 1.97 | (1.82, 2.14)*** | 1.44 | (1.31, 1.58)*** |  |
| Statin |  |  |  |  |  |  |  |  |  |  | 0.678 |
| No | 101271 | 3222226 | 31.43 | 14568 | 345473 | 42.17 | 1.34 | (1.31, 1.36)*** | 1.26 | (1.24, 1.29)*** |  |
| Yes | 18545 | 607585 | 30.52 | 4107 | 105297 | 39.00 | 1.28 | (1.23, 1.32)*** | 1.23 | (1.18, 1.28)*** |  |
| Aspirin |  |  |  |  |  |  |  |  |  |  | 0.1749 |
| No | 112533 | 3690384 | 30.49 | 17293 | 429092 | 40.30 | 1.32 | (1.30, 1.34)*** | 1.26 | (1.23, 1.28)*** |  |
| yes | 7283 | 139426 | 52.24 | 1382 | 21679 | 63.75 | 1.21 | (1.14, 1.28)*** | 1.24 | (1.16, 1.32)*** |  |
|  |  |  |  |  |  |  |  |  |  |  |  |
|  | **Without microvascular disease** | | | **With two microvascular diseases** | | |  |  |  |  | **P for interaction** |
|  | **Event** | **PY** | **IR** | **Event** | **PY** | **IR** | **cHR** | **(95% CI)** | **aHR^#^** | **(95% CI)** |  |
| Overall | 119816 | 3829810 | 31.29 | 11193 | 226171 | 49.49 | 1.58 | (1.55, 1.61)*** | 1.49 | (1.46, 1.53)*** |  |
| Peripheral arterial disease |  |  |  |  |  |  |  |  |  |  | 0.0035 |
| No | 117507 | 3782964 | 31.06 | 10807 | 220155 | 49.09 | 1.58 | (1.55, 1.61)*** | 1.50 | (1.47, 1.53)*** |  |
| Yes | 2309 | 46846 | 49.29 | 386 | 6016 | 64.16 | 1.30 | (1.17, 1.45)*** | 1.28 | (1.13, 1.44)*** |  |
| Insulin use |  |  |  |  |  |  |  |  |  |  | <.0001 |
| No | 118389 | 3768756 | 31.41 | 10378 | 210788 | 49.23 | 1.56 | (1.53, 1.59)*** | 1.48 | (1.45, 1.51)*** |  |
| Yes | 1427 | 61054 | 23.37 | 815 | 15382 | 52.98 | 2.26 | (2.07, 2.46)*** | 1.62 | (1.47, 1.78)*** |  |
| Statin |  |  |  |  |  |  |  |  |  |  | 0.4539 |
| No | 101271 | 3222226 | 31.43 | 8689 | 172165 | 50.47 | 1.60 | (1.57, 1.64)*** | 1.51 | (1.47, 1.54)*** |  |
| Yes | 18545 | 607585 | 30.52 | 2504 | 54005 | 46.37 | 1.52 | (1.46, 1.58)*** | 1.44 | (1.37, 1.51)*** |  |
| Aspirin |  |  |  |  |  |  |  |  |  |  | 0.0007 |
| No | 112533 | 3690384 | 30.49 | 10398 | 214786 | 48.41 | 1.58 | (1.55, 1.62)*** | 1.50 | (1.47, 1.54)*** |  |
| yes | 7283 | 139426 | 52.24 | 795 | 11385 | 69.83 | 1.33 | (1.24, 1.43)*** | 1.36 | (1.25, 1.48)*** |  |
|  |  |  |  |  |  |  |  |  |  |  |  |
|  | **Without microvascular disease** | | | **With three microvascular diseases** | | |  |  |  |  | **P for interaction** |
|  | **Event** | **PY** | **IR** | **Event** | **PY** | **IR** | **cHR** | **(95% CI)** | **aHR^#^** | **(95% CI)** |  |
| Overall | 119816 | 3829810 | 31.29 | 2996 | 47355 | 63.27 | 2.03 | (1.95, 2.10)*** | 1.97 | (1.90, 2.05)*** |  |
| Peripheral arterial disease |  |  |  |  |  |  |  |  |  |  | 0.0900 |
| No | 117507 | 3782964 | 31.06 | 2884 | 45944 | 62.77 | 2.02 | (1.95, 2.10)*** | 1.98 | (1.91, 2.06)*** |  |
| Yes | 2309 | 46846 | 49.29 | 112 | 1411 | 79.38 | 1.62 | (1.34, 1.96)*** | 1.66 | (1.36, 2.04)*** |  |
| Insulin use |  |  |  |  |  |  |  |  |  |  | <.0001 |
| No | 118389 | 3768756 | 31.41 | 2634 | 42505 | 61.97 | 1.98 | (1.90, 2.05)*** | 1.93 | (1.85, 2.01)*** |  |
| Yes | 1427 | 61054 | 23.37 | 362 | 4850 | 74.64 | 3.19 | (2.84, 3.58)*** | 2.25 | (1.98, 2.56)*** |  |
| Statin |  |  |  |  |  |  |  |  |  |  | 0.8902 |
| No | 101271 | 3222226 | 31.43 | 2305 | 35987 | 64.05 | 2.04 | (1.96, 2.13)*** | 1.98 | (1.89, 2.07)*** |  |
| Yes | 18545 | 607585 | 30.52 | 691 | 11368 | 60.79 | 1.99 | (1.85, 2.15)*** | 1.94 | (1.79, 2.11)*** |  |
| Aspirin |  |  |  |  |  |  |  |  |  |  | 0.0076 |
| No | 112533 | 3690384 | 30.49 | 2776 | 44780 | 61.99 | 2.04 | (1.96, 2.11)*** | 1.99 | (1.92, 2.08)*** |  |
| yes | 7283 | 139426 | 52.24 | 220 | 2575 | 85.42 | 1.65 | (1.44, 1.88)*** | 1.72 | (1.49, 1.99)*** |  |

MACE, major adverse cardiovascular events, composite outcome of coronary artery disease, stroke, heart failure or cardiovascular death. PY: Person-Year, IR: Incidence rate, per 1000 persons/years; cHR: crude hazard ratio; aHR: adjusted hazard ratio; CI: confidence interval; aHR#: adjusted for age, sex, obesity, Charlson Comorbidity Index, DCSI score, and medications listed in Table S2 with the Cox proportional hazards regression. * p<0.05, ** p<0.01, *** p<0.001

**Table S4** Characteristics of matched patients with diabetic kidney disease and retinopathy

| **Variables** | **Diabetic kidney disease** | | **Diabetic retinopathy** | | **SMD** |
| --- | --- | --- | --- | --- | --- |
|  | **(N=14651)** | | **(N=14651)** | |  |
|  | **n** | **%** | **n** | **%** |  |
| Sex |  |  |  |  |  |
| female | 6317 | 43.12 | 6356 | 43.38 | 0.005 |
| male | 8334 | 56.88 | 8295 | 56.62 | 0.005 |
| Age |  |  |  |  |  |
| 18-40 | 1565 | 10.68 | 1611 | 11.00 | 0.010 |
| 41-60 | 8454 | 57.70 | 8493 | 57.97 | 0.005 |
| 61-80 | 4632 | 31.62 | 4547 | 31.04 | 0.013 |
| mean, (SD)^a^ | 54.82 | 11.04 | 54.64 | 11.09 | 0.016 |
| Obesity | 250 | 1.71 | 288 | 1.97 | 0.019 |
| Smoking | 284 | 1.94 | 306 | 2.09 | 0.011 |
| Comorbidities |  |  |  |  |  |
| Hypertension | 8112 | 55.37 | 8239 | 56.24 | 0.017 |
| Dyslipidemia | 9426 | 64.34 | 9432 | 64.38 | 0.001 |
| Peripheral arterial disease | 304 | 2.07 | 328 | 2.24 | 0.011 |
| COPD | 1673 | 11.42 | 1753 | 11.97 | 0.017 |
| Alcohol-related disorders | 258 | 1.76 | 271 | 1.85 | 0.007 |
| Liver cirrhosis | 190 | 1.30 | 216 | 1.47 | 0.015 |
| Connective tissue diseases | 184 | 1.26 | 196 | 1.34 | 0.007 |
| Cancers | 505 | 3.45 | 540 | 3.69 | 0.013 |
| Psychosis | 510 | 3.48 | 508 | 3.47 | 0.001 |
| Depression | 141 | 0.96 | 152 | 1.04 | 0.008 |
| Dementia | 52 | 0.35 | 49 | 0.33 | 0.003 |
| CCI |  |  |  |  |  |
| 1 | 12557 | 85.71 | 12475 | 85.15 | 0.016 |
| 2-3 | 1753 | 11.97 | 1809 | 12.35 | 0.012 |
| >3 | 341 | 2.33 | 367 | 2.50 | 0.012 |
| Medications |  |  |  |  |  |
| Antidiabetic drugs |  |  |  |  |  |
| Metformin | 10698 | 73.02 | 10704 | 73.06 | 0.001 |
| Sulfonylurea | 7432 | 50.73 | 7464 | 50.95 | 0.004 |
| Meglitinides | 599 | 4.09 | 608 | 4.15 | 0.003 |
| Thiazolidinedione | 735 | 5.02 | 770 | 5.26 | 0.011 |
| DPP-4 inhibitors | 2159 | 14.74 | 2247 | 15.34 | 0.017 |
| AGI | 838 | 5.72 | 869 | 5.93 | 0.009 |
| SGLT2 inhibitors | 79 | 0.54 | 78 | 0.53 | 0.001 |
| Number of oral antidiabetic drugs | |  |  |  |  |
| 1 | 6582 | 44.93 | 6451 | 44.03 | 0.018 |
| 2-3 | 7811 | 53.31 | 7942 | 54.21 | 0.018 |
| >3 | 258 | 1.76 | 258 | 1.76 | 0.000 |
| GLP-1 RA | 23 | 0.16 | 28 | 0.19 | 0.008 |
| Insulins | 1229 | 8.39 | 1267 | 8.65 | 0.009 |
| Antihypertensive drugs |  |  |  |  |  |
| ACEI/ARB | 4574 | 31.22 | 4747 | 32.40 | 0.025 |
| β-blockers | 191 | 1.30 | 223 | 1.52 | 0.019 |
| Calcium-channel blockers | 2636 | 17.99 | 2665 | 18.19 | 0.005 |
| Diuretics | 868 | 5.92 | 852 | 5.82 | 0.005 |
| Number of antihypertensive drugs | |  |  |  |  |
| 1 | 12706 | 86.72 | 12631 | 86.21 | 0.015 |
| 2-3 | 1940 | 13.24 | 2016 | 13.76 | 0.015 |
| >3 | 5 | 0.03 | 4 | 0.03 | 0.004 |
| Statin | 4240 | 28.94 | 4357 | 29.74 | 0.018 |
| Aspirin | 809 | 5.52 | 808 | 5.52 | 0.000 |

Data are shown as n (%) or mean ± SD

Abbreviations: SMD, standardized mean difference; COPD, chronic obstructive pulmonary disease; CCI, Charlson Comorbidity Index; DPP-4, Dipeptidyl peptidase-4; AGI, Alpha-glucosidase inhibitors; SGLT2, sodium-glucose cotransporter 2; GLP-1RA, glucagon-like peptide-1 receptor agonist; ACEI, angiotensin converting enzyme inhibitors; ARB, angiotensin receptor blockers. A SMD < 0.1 indicates a negligible difference between the two groups of patients

^a^: Student’s t-test

**Table S5** Characteristics of matched patients with diabetic kidney disease and neuropathy

| **Variables** | **Diabetic kidney disease** | | **Diabetic neuropathy** | | **SMD** |
| --- | --- | --- | --- | --- | --- |
|  | **(N=24996)** | | **(N=24996)** | |  |
|  | **n** | **%** | **n** | **%** |  |
| Sex |  |  |  |  |  |
| Female | 10596 | 42.39 | 10667 | 42.67 | 0.006 |
| Male | 14400 | 57.61 | 14329 | 57.33 | 0.006 |
| Age |  |  |  |  |  |
| 18-40 | 2987 | 11.95 | 2880 | 11.52 | 0.013 |
| 41-60 | 13986 | 55.95 | 14116 | 56.47 | 0.010 |
| 61-80 | 8023 | 32.10 | 8000 | 32.01 | 0.002 |
| Mean, (SD)^a^ | 54.76 | 11.73 | 54.84 | 11.62 | 0.007 |
| Obesity | 699 | 2.80 | 694 | 2.78 | 0.001 |
| Smoking | 681 | 2.72 | 681 | 2.72 | 0.000 |
| Comorbidities |  |  |  |  |  |
| Hypertension | 14398 | 57.60 | 14644 | 58.59 | 0.020 |
| Dyslipidemia | 17244 | 68.99 | 17258 | 69.04 | 0.001 |
| Peripheral arterial disease | 593 | 2.37 | 536 | 2.14 | 0.015 |
| COPD | 4062 | 16.25 | 4163 | 16.65 | 0.011 |
| Alcohol-related disorders | 740 | 2.96 | 733 | 2.93 | 0.002 |
| Liver cirrhosis | 520 | 2.08 | 502 | 2.01 | 0.005 |
| Connective tissue diseases | 538 | 2.15 | 529 | 2.12 | 0.002 |
| Cancers | 1294 | 5.18 | 1260 | 5.04 | 0.006 |
| Psychosis | 1255 | 5.02 | 1256 | 5.02 | 0.000 |
| Depression | 366 | 1.46 | 368 | 1.47 | 0.001 |
| Dementia | 178 | 0.71 | 182 | 0.73 | 0.002 |
| CCI |  |  |  |  |  |
| 1 | 21462 | 85.86 | 21424 | 85.71 | 0.004 |
| 2-3 | 2853 | 11.41 | 2895 | 11.58 | 0.005 |
| >3 | 681 | 2.72 | 677 | 2.71 | 0.001 |
| Medications |  |  |  |  |  |
| Oral antidiabetic drugs |  |  |  |  |  |
| Metformin | 14968 | 59.88 | 15056 | 60.23 | 0.007 |
| Sulfonylurea | 9512 | 38.05 | 9675 | 38.71 | 0.013 |
| Meglitinides | 755 | 3.02 | 778 | 3.11 | 0.005 |
| Thiazolidinedione | 892 | 3.57 | 914 | 3.66 | 0.005 |
| DPP-4 inhibitors | 3009 | 12.04 | 2998 | 11.99 | 0.001 |
| AGI | 1084 | 4.34 | 1081 | 4.32 | 0.001 |
| SGLT2 inhibitors | 139 | 0.56 | 127 | 0.51 | 0.007 |
| Number of oral antidiabetic drugs | |  |  |  |  |
| 1 | 14578 | 58.32 | 14439 | 57.77 | 0.011 |
| 2-3 | 10150 | 40.61 | 10276 | 41.11 | 0.010 |
| >3 | 268 | 1.07 | 281 | 1.12 | 0.005 |
| GLP-1 RA | 32 | 0.13 | 30 | 0.12 | 0.002 |
| Insulins | 1503 | 6.01 | 1544 | 6.18 | 0.007 |
| Antihypertensive drugs |  |  |  |  |  |
| ACEI/ARB | 7351 | 29.41 | 7263 | 29.06 | 0.008 |
| β-blockers | 459 | 1.84 | 466 | 1.86 | 0.002 |
| Calcium-channel blockers | 4439 | 17.76 | 4583 | 18.33 | 0.015 |
| Diuretics | 1106 | 4.42 | 1105 | 4.42 | 0.000 |
| Number of antihypertensive drugs | |  |  |  |  |
| 1 | 22012 | 88.06 | 21958 | 87.85 | 0.007 |
| 2-3 | 2979 | 11.92 | 3031 | 12.13 | 0.006 |
| >3 | 5 | 0.02 | 7 | 0.03 | 0.005 |
| Statin | 6432 | 25.73 | 6356 | 25.43 | 0.007 |
| Aspirin | 1278 | 5.11 | 1291 | 5.16 | 0.002 |

Data are shown as n (%) or mean ± SD

Abbreviations: SMD, standardized mean difference; COPD, chronic obstructive pulmonary disease; CCI, Charlson Comorbidity Index; DPP-4, Dipeptidyl peptidase-4; AGI, Alpha-glucosidase inhibitors; SGLT2, sodium-glucose cotransporter 2; GLP-1RA, glucagon-like peptide-1 receptor agonist; ACEI, angiotensin converting enzyme inhibitors; ARB, angiotensin receptor blockers. A SMD < 0.1 indicates a negligible difference between the two groups of patients

^a^: Student’s t-test

**Table S6** Characteristics of matched patients with diabetic retinopathy and neuropathy

| **Variables** | **Diabetic retinopathy** | | **Diabetic neuropathy** | | **SMD** |  |
| --- | --- | --- | --- | --- | --- | --- |
|  | **(N=13250)** | | **(N=13250)** | |  |  |
|  | **n** | **%** | **n** | **%** |  |  |
| Sex |  |  |  |  |  |  |
| female | 5923 | 44.70 | 5706 | 43.06 | 0.033 |  |
| male | 7327 | 55.30 | 7544 | 56.94 | 0.033 |  |
| Age |  |  |  |  |  |  |
| 18-40 | 1454 | 10.97 | 1451 | 10.95 | 0.001 |  |
| 41-60 | 7676 | 57.93 | 7696 | 58.08 | 0.003 |  |
| 61-80 | 4120 | 31.09 | 4103 | 30.97 | 0.003 |  |
| mean, (SD)^a^ | 54.65 | 11.08 | 54.66 | 11.05 | 0.000 |  |
| Obesity | 249 | 1.88 | 238 | 1.80 | 0.006 |  |
| Smoking | 255 | 1.92 | 249 | 1.88 | 0.003 |  |
| Comorbidities |  |  |  |  |  |  |
| Hypertension | 7299 | 55.09 | 7075 | 53.40 | 0.034 |  |
| Dyslipidemia | 8314 | 62.75 | 8319 | 62.78 | 0.001 |  |
| Peripheral arterial disease | 256 | 1.93 | 230 | 1.74 | 0.015 |  |
| COPD | 1553 | 11.72 | 1499 | 11.31 | 0.013 |  |
| Alcohol-related disorders | 213 | 1.61 | 198 | 1.49 | 0.009 |  |
| Liver cirrhosis | 175 | 1.32 | 149 | 1.12 | 0.018 |  |
| Connective tissue diseases | 166 | 1.25 | 162 | 1.22 | 0.003 |  |
| Cancers | 466 | 3.52 | 431 | 3.25 | 0.015 |  |
| Psychosis | 421 | 3.18 | 381 | 2.88 | 0.018 |  |
| Depression | 128 | 0.97 | 120 | 0.91 | 0.006 |  |
| Dementia | 39 | 0.29 | 33 | 0.25 | 0.009 |  |
| CCI |  |  |  |  |  |  |
| 1 | 11403 | 86.06 | 11490 | 86.72 | 0.019 |  |
| 2-3 | 1569 | 11.84 | 1500 | 11.32 | 0.016 |  |
| >3 | 278 | 2.10 | 260 | 1.96 | 0.010 |  |
| Medication |  |  |  |  |  |  |
| Oral Antidiabetic drugs (OAD) | 9763 | 73.68 | 9879 | 74.56 | 0.020 |  |
| Metformin | 6861 | 51.78 | 6912 | 52.17 | 0.008 |  |
| Sulfonylurea | 547 | 4.13 | 514 | 3.88 | 0.013 |  |
| Meglitinides | 741 | 5.59 | 727 | 5.49 | 0.005 |  |
| Thiazolidinedione | 1927 | 14.54 | 1898 | 14.32 | 0.006 |  |
| DPP-4 inhibitors | 805 | 6.08 | 767 | 5.79 | 0.012 |  |
| AGI | 56 | 0.42 | 54 | 0.41 | 0.002 |  |
| SGLT2 inhibitors |  |  |  |  |  |  |
| Number of oral antidiabetic drugs | |  |  |  |  |  |
| 1 | 5778 | 43.61 | 5757 | 43.45 | 0.003 |  |
| 2-3 | 7215 | 54.45 | 7238 | 54.63 | 0.003 |  |
| >3 | 257 | 1.94 | 255 | 1.92 | 0.001 |  |
| GLP-1 RA | 22 | 0.17 | 22 | 0.17 | 0.000 |  |
| Insulins | 1073 | 8.10 | 1069 | 8.07 | 0.001 |  |
| Antihypertensive drugs |  |  |  |  |  |  |
| ACEI/ARB | 4145 | 31.28 | 3941 | 29.74 | 0.033 |  |
| β-blockers | 192 | 1.45 | 169 | 1.28 | 0.015 |  |
| Calcium-channel blockers | 2422 | 18.28 | 2273 | 17.15 | 0.029 |  |
| Diuretics | 743 | 5.61 | 668 | 5.04 | 0.025 |  |
| Number of antihypertensive drugs | |  |  |  |  |  |
| 1 | 11463 | 86.51 | 11613 | 87.65 | 0.034 |  |
| 2-3 | 1782 | 13.45 | 1633 | 12.32 | 0.034 |  |
| >3 | 5 | 0.04 | 4 | 0.03 | 0.004 |  |
| Statin | 3859 | 29.12 | 3827 | 28.88 | 0.005 |  |
| Aspirin | 737 | 5.56 | 720 | 5.43 | 0.006 |  |

Data are shown as n (%) or mean ± SD

Abbreviations: SMD, standardized mean difference; COPD, chronic obstructive pulmonary disease; CCI, Charlson Comorbidity Index; DPP-4, Dipeptidyl peptidase-4; AGI, Alpha-glucosidase inhibitors; SGLT2, sodium-glucose cotransporter 2; GLP-1RA, glucagon-like peptide-1 receptor agonist; ACEI, angiotensin converting enzyme inhibitors; ARB, angiotensin receptor blockers., standardized A SMD < 0.1 indicates a negligible difference between the two groups of patients

^a^: Student’s t-test
